# Gene expression patterns within cell lines are predictive of chemosensitivity

**DOI:** 10.1186/1471-2164-9-74

**Published:** 2008-02-08

**Authors:** Brian Z Ring, Stella Chang, L Winston Ring, Robert S Seitz, Douglas T Ross

**Affiliations:** 1 Applied Genomics Inc., Burlingame, CA, 94010, USA; 2 Juvaris BioTherapeutics, Burlingame, CA, 94010, USA; 3 Applied Genomics Inc., Huntsville, AL, 35806, USA

## Abstract

**Background:**

The NCI has undertaken a twenty-year project to characterize compound sensitivity patterns in a selected set of sixty tumor derived cell lines. Previous studies have explored the relationship between compound sensitivity patterns to gene expression, protein expression, and DNA copy number for these same cell lines. A strong correlation between the pattern of expression of a biomarker and sensitivity to a compound could suggest a clinically interesting biological relationship between the two.

**Results:**

We isolated RNA's and measured expression of 40000 genes using cDNA microarrays from the fifty-nine publicly available cell lines. Analysis of this data set in comparison with published gene expression data sets demonstrates a high degree of reproducibility in expression level measurements even using completely independent RNA preparations and array technologies. Using the fifty-nine cell lines for discovery and an additional seven cell lines for which extensive compound sensitivity data were available as a test set, we determined that gene-compound pairs with a correlation coefficient above 0.6 had a false discovery rate of approximately 5%. Large scale features of the gene expression and chemosensitivity data, such as tissue of origin and other physiological factors, did not seem to explain the majority of correlations between gene and compound patterns.

**Conclusion:**

A comparison of gene expression and compound sensitivity in panels of cell lines was demonstrated to have a relatively high validation and low false discovery rate supporting the use of this approach and datasets for identifying candidate biomarkers and targeted biologically active compounds.

## Background

The ability to predict the sensitivity of tumors to chemotherapy is a critical element in the drug discovery process, yet means of rationally realizing this objective early in the drug development process are limited. Despite many recent advances, cancer drug discovery remains an extremely expensive and failure prone endeavor when compared to success rates in other areas of drug development [1,2]. The US National Cancer Institute's (NCI) Developmental Therapeutics Program (DTP) screen for anticancer drugs has been a systematic attempt to explore the functional relationship between chemical compounds and toxicity in cell line cancer models, and was one of the first large scale public efforts to identify novel drugs for solid tumors (for review, see [3]). The DTP screen has made publicly available growth inhibition data on sixty tumor derived cell lines (the NCI60) for tens of thousands of compounds. The coupling of this effort with a genomics perspective, in which the basal level of expression of genes on the NCI60 panel of cells was measured, introduced a novel approach for establishing a link between new therapies and biomarker candidates assessed by gene and protein expression patterns [4-8]. This pharmcogenomics approach supposed that genes with patterns of expression across the cell lines which are highly correlated with compound sensitivity are candidate clinical biomarkers of drug efficacy, potentially even direct effectors of drug action, or targets for novel drug development with existing compound leads.

Known relationships between genes and drugs have been re-identified via this approach using the NCI60 cell lines and other cell line panels, as well as novel targeting of drugs based on these studies, are being evaluated (e.g. [4,5,9-14]). Relationships identified from these screens have been supported by chemical knowledge about the compounds, suggesting that a rational relationship may exist between compound structure, mechanism of action, and determinants of cell toxicity as assessed by gene expression pattern [11,15-19]. The first study to compare a large scale gene expression study with the NCI60 data found that the antimetabolite 5-FU was negatively correlated with dihydropyrimidine dehydrogenase [4]. As this is the rate-limiting enzyme in 5-FU catabolism, high enzyme levels would be expected to reduce exposure of cells to the active forms of 5-FU. Another study employed a set of cell lines specifically chosen for resistance or sensitivity to doxorubicin and a multigene model was trained upon the expression data from these lines to identify gene expression patterns indicative of resistance to this drug [9]. This model was shown to significantly classify doxorubicin-treated breast cancer patients into distinct prognostic classes. A more extensive study used NCI60 gene expression data to identify gene expression signatures that predict sensitivity to several compounds, and validated these signatures in an independent set of cell lines [14]. The question still remains as to the overall efficacy of the approach as a useful early discovery tool in pharmacological research.

To assess the validity and false discovery rate in these pharmacogenomic associations we measured the expression of 40,000 genes in the fifty nine publicly available NCI60 cell lines using spotted cDNA microarrays and measured the expression of these genes on an additional seven lines also employed in the DTP screen. Using the available growth inhibition (GI50) data for 41000 compounds, we calculated Spearman correlations for all pairs of gene expression and compound sensitivity patterns and assessed the ability of relationships nominated in the NCI60 to predict relationships in the test set of seven cell lines. This allowed us to gauge the likelihood that relationships found between drugs and genes in this chemical genomic approach are reproducible and worth pursing with more targeted studies. This data is available in the Gene Expression Omnibus, accession number GSE7947.

## Methods

### Measurements of gene expression

The microarray studies were performed with cDNA spotted arrays produced by collaborators at Stanford University, as previously described [6]. Briefly, forty-thousand cDNA clones were PCR amplified and the PCR products printed on treated glass microscope slides. Cell lines were grown from the publicly available NCI DTP frozen stocks (see Additional file 1) in RPMI-1640 supplemented with phenol red, glutamine (2 mM) and 5% fetal calf serum. To minimize the contribution of variations in culture conditions or cell density to differential gene expression, we grew each cell line to 80% confluence and isolated mRNA 24 hours after transfer to fresh medium. Cells were lysed in buffer containing Protein/Rnase Degrader (Invitrogen) and messenger RNA was purified with the FastTrack 2.0 purification kit (Invitrogen). For each comparative array hybridization, labeled cDNA was synthesized by reverse transcription from test cell mRNA in the presence of Cy5-dUTP, and from the reference mRNA with Cy3-dUTP, using the Superscript II reverse-transcription kit (Gibco-BRL), hybridized to microarray at 65°C overnight. The reference sample was derived from eleven of the fifty nine cell lines. After hybridization, each microarray was washed, then scanned using GenePix 4000A microarray scanner (Axon Instruments, Union City, CA).

Initial data analysis was carried out using GenePix Pro 3.0 (Axon Instruments). The new array data presented here as well as the previous Stanford arrays were quality controlled to manually flag and exclude apparent problematic spots. All non-flagged array elements for which the fluorescent intensity in each channel was at least 300 units and the regression correlation between the red and green channels was greater than 0.6 were considered well measured and included in the study (see Additional file 2). Inclusion of a gene in correlative studies required that greater than 80% of measurements across all the cell lines in the study were present. Other expression data sets used in these analyses were also subject to this filter. Array information from Genomics Institute of the Novartis Research Foundation (GNF) employed only measurements with a 'present' call when absent/present calls were available [20,21]. Additionally, the GNF arrays were filtered for the probes that gave a coefficient of variation (the standard deviation divided by the average) greater than 0.1.

### NCI60 chemosensitivity data

The chemosensitivity assays of the NCI have been previously described [5,22]. Briefly, the cell lines were grown in 96-well plates and exposed to the test compound for 48 hours. Growth inhibition is expressed in terms of the GI50, the concentration required to inhibit cell growth by 50% in comparison with untreated controls. The data was filtered to include only compounds that had data in greater than 75% of cell lines and which exceeded a minimal level of variance across the lines (a standard deviation of the ranked data greater than 0.1 across the available lines). The August 2004 release of the GI50 data from the DTP was used for these analyses (see Additional file 3).

### Statistical methods

Both Spearman rank correlation and Pearson correlations were assessed as a method for comparing the GI50 and gene expression data. Spearman rank correlation was chosen as it accommodated better the constricted range and dynamic limits of the GI50 measurements. Correlations were only measured when drug and gene data were both present for 75% of all lines. The average number of contributing cell lines to a correlation measurement in the data sets was 49, and this figure was used in determining degrees of freedom for other calculations. As the number of correlate pairs measured was nearly 300 million, only correlations with an absolute value greater than 0.5 were included in most analyses (see Additional file 4).

The false discovery rate for each gene:drug correlate pairs was estimated as a q value using software created by Alan Dabney and John Storey [23,24]. The *q*-value for a particular correlation estimates the proportion, on average, of incurred false positives for correlations with a similar level of significance. The number of correlate pairs measured in this analysis was too many to readily assess for estimation of q values, so q values were estimated from ten random samplings of the gene expression and GI50 data sets. All gene:drug correlates were measured from these samplings with all correlate pairs retained (not only those with an |R| ≥ 0.50 as in the main study), using 155000 correlation pairs in total.

The identified correlations between gene expression and compound sensitivity can be due to broad patterns between the cell lines based on their tissues of origin or other commonly shared physiological features. Principal component analysis was used to identify patterns in both the gene and drug sensitivity datasets using the princomp() function of S-Plus ver. 6.2. These and other higher level features of the datasets such as tissue of origin gene expression patterns were compared via Pearson correlations.

## Results

### Reproducibility

Measurement of gene expression with microarrays is now an accepted laboratory tool, but the plethora of technologies, experimental approaches, and other technical details have continued to raise questions about the reliability of array based measurements of gene expression [25]. The existence of four datasets of gene expression, measured using three independent preparation of RNA from 59 cell lines provides an opportunity for perhaps the largest study of the overall reproducibility of both the biologic stability of gene expression under cell culture conditions as well as the precision of measurements using gene array technology. To assess the reproducibility of the gene expression data reported here, we compared the results from three different published NCI60 cell line gene expression data sets and the new data reported herein. As a metric of the reproducibility of the data, we compared the patterns of gene expression across the sixty cell lines from replicate measurements of individual genes. These replications were either from independent array elements within arrays or measurements of the same gene compared across datasets.

**Figure 1:**
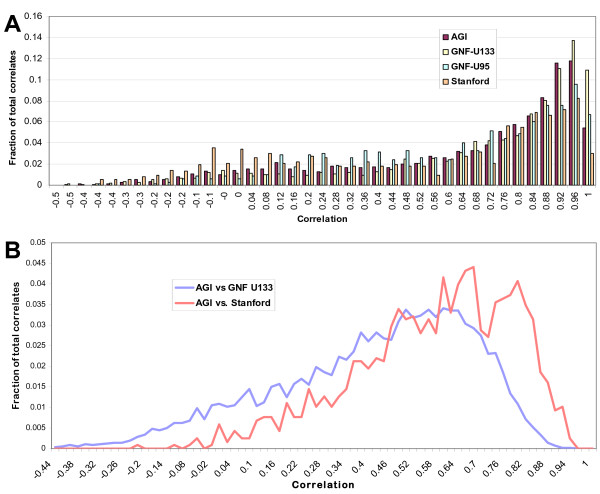
Replication of gene expression studies. A) Distribution of correlations between duplicates within studies. Array spots containing duplicate genes (identical Unigene ID's) within the different microarray assays were compared by Pearson correlations. The four array experiments shown have comparable levels of reproducibility. For the AGI expression data, over half of duplicated array spots showed a Pearson correlation of 0.8 or greater, and over 93% of duplicates had a positive correlation. B) Distribution of correlations between duplicates between studies. Array spots containing duplicate genes between the different microarray assays were compared by Pearson correlations. The expression measurements showed 99% of duplicate genes have a positive correlation between the AGI arrays and the similarly spotted-cDNA Stanford arrays between their patterns of expression across the cell lines. Comparison to the GNF U133 printed array study showed 93% of duplicated genes have a positive correlation.

As expected, the comparison of replicates measured on the same array showed the greatest reproducibility (Fig. 1A); most genes showed a highly conserved pattern of gene expression across the cell lines. Over half of genes represented by multiple independent clones showed a Pearson correlation between array elements of 0.8 or greater, and over 93% of duplicated genes had a positive correlation.

This high conservation of expression level was also found when compared within other expression studies using the NCI60 cell lines, including both the Stanford spotted cDNA arrays and the GNF's Affymetrix GeneChip Human Genome U133A and U95 printed arrays (Fig. 1A). This suggests that measurements of gene expression are reproducible across array platforms. Comparison of expression patterns of genes between arrays also showed a high degree of conservation of expression, despite that these assays entailed the use of different RNA preparations as well as different array technologies (Fig. 1B). The expression measurements of the NCI60 lines when compared to those of the similar spotted Stanford cDNA array study showed 99% of duplicate genes have a positive correlation between their patterns of expression across the cell lines. Comparison to the printed GNF U133 array study showed 93% of duplicated genes have a positive correlation.

### False discovery rate

One significant utility of these large gene and compound sensitivity datasets is the potential that the co-variation of compound sensitivity and gene expression across the sixty cell lines will reflect either functional relationships or identify biomarker candidates that are associated with compound sensitivity. While several other studies have shown examples of empirically validated gene:drug associations nominated in similar analyses, we wanted to assess our overall confidence in the likelihood that significant correlations would not be spurious false positives. This is especially significant when assessing the approximately 300 million correlations derived from analyses such as this and assessing whether this approach is a valid early discovery tool. Applying a standard Bonferroni alpha correction to this number of multiple hypotheses generated by calculating correlations between gene expression and compound sensitivity patterns would suggest that only correlations above ~0.75 are likely to be valid (df = 47, with 297 million hypotheses). Furthermore, a Bonferroni correction assumes that the associations are independent of one another, which will not be the case among all the gene expression and GI50 measurements. A strict correction of this size is far too conservative, but it remains untested what scale of correction is needed to address the large number of comparisons being made. Similarly, it thus also remains untested whether this approach to identifying associations betweens genes and prospective drugs produces a useful number of reproducible candidates for further study.

**Figure 2:**
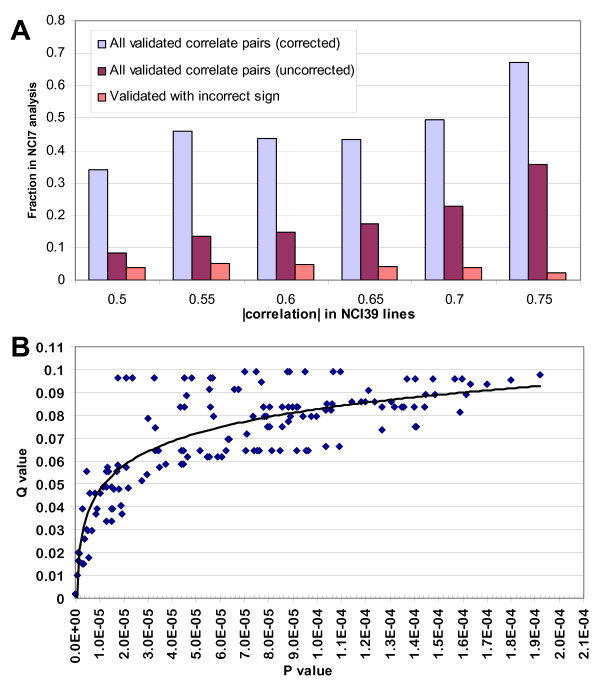
Estimation of the false discovery rate. A) Validation of NCI39 derived gene:drug correlate pairs in the NCI7 cell lines. The percent of pairs which validated increased with the increasing correlation coefficient of the nominated gene-drug relationship in the NCI39 training set, and very few candidates were found to have significant but oppositely signed correlations in the NCI7 data. When the percentage of validated correlates is adjusted by the power of a correlation, assuming a single-sided p value of 0.5 is considered significant with a sample size of six, it becomes apparent that a majority of the gene:drug correlate pairs with correlations greater than 0.7 would have validated in a replication of the original experiment. B) Comparison of q-values and p-values for the gene:drug correlates. To estimate the q value in these very large data sets, random subsets of the gene expression and GI50 data were iteratively compared and the distribution of p values were measured for ~150000 correlations in total. A q value of 0.05 (i.e., that for every 100 significant correlates, five false correlates are expected.) is associated with a p value of approximately 1 × 10^-5^.

In order to quantitatively assess the reproducibility of the gene:drug correlates, we measured gene expression on seven additional cell lines (termed herein the 'NCI7', see Additional file 1) for which compound sensitivity data was available for several thousand compounds from the DTP. We then determined whether candidate correlate pairs nominated in the NCI60 would predict relationships between genes and compounds in this test set of cell lines. As these seven cell lines do not originate from the full panel of tumor types found in the NCI60 cell lines, we used only those thirty-nine cell lines whose tissue type were represented in the additional seven cell lines (the 'NCI39'). Figure 2A depicts the percent of candidate correlates from the NCI39 that also showed significant correlations (two sided p < 0.05) across the NCI7. Although an analysis of only seven cell lines is underpowered, only 15% of gene:drug pairs with a correlation of 0.6 or greater have a significant but incorrectly signed result in the NCI7 analysis. Defining validation as a significant correlation between gene and compound patterns, the percent validated increased with the increasing correlation coefficient of the nominated gene-drug relationship in the NCI39 training set. When the degree that the analysis was underpowered is addressed it becomes apparent that a majority of gene:drug correlate pairs with correlations greater than 0.7 would likely be reproducible in a study similar to the original analysis using 60 cell lines. This estimate was accomplished by dividing the percentage of validated pairs within a range of measured correlations by the power of the study assuming a single-sided p value of 0.05 is considered significant (assuming that, on average, six cell lines contributed to the correlations in the NCI7 study). This degree of expected validation far exceeds that which would be expected if a Bonferroni correction were appropriate, which would suggest that only correlations greater than 0.84 (df = 32) are likely to be significant. A corresponding degree of significance using the full NCI60 panel suggests that correlations greater than 0.6 are likely to be reproducible (Table 1).

**Table 1:** Comparison of q and p values for the NCI60 and NCI39 studies. The NCI7 study suggested that the majority of correlates pairs identified in the NCI39 study with a correlation greater than 0.7 were reproducible. This correlation is associated with a q value of 0.05. A similar q value in the NCI60 study is associated with a correlation of just under 0.60. This assessment of acceptable correlations for gene:drug correlates worthy of potential further study is far lower than a conventional correction for multiple hypotheses would suggest.

	**p value**	**Bonferroni corrected p-value**	**q value**	**Correlation**
**'NCI39'**	2.4 × 10-4	1	0.2	0.59
	2 × 10-5	1	0.1	0.66
	5.7 × 10-6	1	0.05	0.7
	1.1 × 10-10	0.05	nd	0.84
**NCI60**	~2 × 10-4	1	0.1	0.5
	1.2 × 10-5	1	0.05	0.57
	5.7 × 10-6	1	0.05	0.59
	1.1 × 10-10	0.05	nd	0.75

Novel approaches to estimating the false discovery rate have been recently employed in large genomic studies [23,26,27]. As a second estimate of the error rate of the gene-compound correlates, we determined a false discovery rate, as estimated by a q-value, for each correlate. The q-value measures the predicted false discovery rate associated with a significant test when multiple hypotheses are tested, i.e. a q-value of 0.05 implies that for every 100 significant correlates, five false correlates are expected. A comparison of multiple false discovery estimations on several gene expression data sets suggests that the q-value method has a high apparent power and strong control of the FDR [28,29]. Analysis of this predicted false discovery rate is consistent in this experiment with the NCI7 validation of the NCI39 analysis. To estimate the q value in these very large data sets, random subsets of the gene expression and GI50 data were iteratively compared and the distribution of p values were measured for ~150000 correlations in total for both the NCI60 study (Fig. 2B) and the NCI39 study (data not shown). In the NCI39 dataset a p value of 5.74 × 10-6 has a corresponding q value of 0.05, which on average was associated with a correlation of 0.7. This estimation of the false discovery rate roughly corresponds to the distribution of NCI39 correlates found to be significant yet incorrectly signed in the NCI7 test. For a screen employing the full sixty cell lines this analysis would indicate that a q-value of 0.05 correlates with a correlation coefficient of at least 0.57, far lower than the correlation cutoff of 0.75 suggested by a standard alpha correction (Table 1). The NCI7 validation study and the q value estimation of the false discovery rate both suggest that correlations with an absolute value greater than 0.6 are very likely to be reproducible.

### Identification of large-scale features in the gene expression and compound sensitivity data

A potential limitation in the cross correlation of data sets, especially in regards to the hope that gene:drug correlates may indicate functional relationships, is that large scale features of the gene expression and or chemosensitivity data- such as tissue of origin and other physiological factors, may drive many of the identified correlations. The comparison of the data sets only establishes correlation, not causation, and if many of the correlations are the result of relatively non-specific factors, or the causative gene(s) co-varies with many others in the dataset, large gene expression patterns reflecting physiology unresolved by the sampling of cell lines in the NCI60 may obscure causative gene:drug correlations. If so, this could greatly decrease the likelihood that identified genes could be potential novel and specific effectors of drug action, and also diminish the chance that identified associations would translate into clinically useful aids in directing therapies to susceptible tumors.

**Figure 3:**
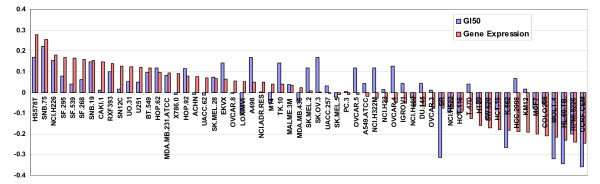
Comparison of mesenchymal-like components between the compound sensitivity and gene expression data sets. Principle component analysis revealed a shared feature in each data set. The second component of the gene expression data, containing 8.4% of the variance, was largely defined by a panel of cell lines identified previously as potentially related to mesenchymal differentiation. The 'mesenchymal' component also appeared to be a significant features of the GI50 data, the cell lines ordered here as in the plot of the gene expression data, though this component explained only 0.9% of the variance. The two 'mesenchymal' components of the gene expression and drug data correlate with each other with an R of 0.62, and therefore represent a strong but readily identifiable correlation. Thus identified, correlations with similarities to these patterns can be noted and subtracted or filtered from analyses.

Major features in the gene expression and growth inhibition data sets can be identified through principal components analysis. The first principal component in each data set primarily reflected the strong pattern associated with the leukemia cell lines (possibly due to their suspended, rather than adherent, state) which was also present in the first component of the GI50 data. The second component was largely defined by a panel of cell lines identified previously from gene expression studies as sharing a molecular physiology related to mesenchymal differentiation [6], and contained 8.4% of the variance of the gene expression data, thus explaining a minor yet significant amount of the variation in the expression patterns. The third component of the expression data was dominated by the melanoma and leukemia lines' expression, explaining 5.2% of the variance. The leukemia and 'mesenchymal' components of the data sets also appear to be the most significant features that explain the variance of the GI50 data, though the second 'mesenchymal' component explained only 0.9% of the variance. The two 'mesenchymal' components of the gene expression and drug data correlate with each other with an R of 0.62, and therefore represent a strong but readily identifiable correlation (Fig. 3). Thus identified, correlations with similarities to these patterns can be noted and subtracted or filtered from analyses, if desired [30]. As might be expected given the now readily apparent diversity that exists within tumors arising in single organs, efforts to assess whether tissue-of-origin contributed towards correlations between genes and drugs failed to identify a strong driver of identified associations (data not shown).

### Confirmation of known gene:drug associations

Another measure of the reproducibility of the assay is the ability to repeat outcomes of similar analyses or associations between genes and drugs already established in the literature. As an assessment of the global validity of the analysis such examples have limited utility, but do serve as an important confirmation that anticipated findings are observed. For example, in this analysis expression of epidermal growth factor receptor (EGFR) correlated with sensitivity (R = 0.77) to TP4EK (NSC 676497), a recombinant exotoxin targeted to the EGFR through TGFα as previously noted by K. Wosikowski, et al. using RTPCR expression measurements on the NCI60 cell lines [31]. ErbB2 expression correlated with sensitivity (R = 0.60) to an anti-ErbB2 antibody fragments with endotoxin payload (NSC 683039). MDR is associated with resistance to 38 compounds with an R<-0.5, and MRP1 is associated with resistance to 279 compounds with an R<-0.5. Members of the MDR and MRP subfamilies are involved in multidrug resistance; the proteins encoded by these genes are ATP-dependent drug pumps for xenobiotic compounds with broad substrate specificity, and it has been previously observed that the NCI60 lines expressing MDR have resistance to a large panel of drugs [32]. On average in the set of genes employed in this analysis there are four compounds negatively correlated per gene, so the high number of compounds negatively correlated with MDR1 and MRP1 are in accord with the well-established role of these proteins in providing resistance to a variety of compounds.

## Discussion and Conclusion

Drug discovery in the field of oncology has proven to be an expensive and arduous task, with one of the lowest success rates at the clinical trial level of validation (5%, from phase I through registration, compared to an average of 11% in all therapeutic areas) [1,2]. A better means of identifying robust candidates early in the drug discovery process is clearly needed. A promising genomic approach to this problem uses gene expression patterns measured across the genome for dozens of cell lines compared to patterns of chemosensitivity drug panels measured across the same cell lines. A comparison of the tens of thousands of compound sensitivity profiles from the Developmental Therapeutics Program of the NCI with genomic scale measurements of gene expression across the NCI60 cell lines creates the potential for a novel systematic approach for the early identification of compound specific biomarker candidates and potential targeted lead candidates for the treatment of solid tumors.

Though a number of studies have employed this approach to identify candidate gene:drug pairs for further study, it has yet to be established the overall efficacy of this method. While there is a growing number of reports which suggest that association of gene expression patterns in cell line studies can identify candidate gene:drug pairs of potential clinical importance, it hasn't been established how efficient is this analytical approach. Herein we show that gene and drug correlates identified in the NCI60 data validate at a much higher rate on a set of seven independent cell lines than a standard Bonferroni alpha correction would predict. The rate of validation empirically measured with the NCI7 lines was consistent with the q-value analysis, used as an estimate of the false discover rate, which suggested that Spearman correlates with an absolute value above a threshold of 0.57 have false discovery frequency of less than 0.05 in this study. Other cell line studies looking at relationships between gene expression and compound sensitivity have suggested arbitrarily stringent criteria for identifying correlations that warrant further study or have used a standard measurement of significance with no alpha correction (e.g., [12,18]. This study would suggest that both approaches may be improved upon, generating an optimum number of results with a high confidence of reproducibility. A correlation of approximately 0.6 in the studies presented here results in over 99.9% of correlate pairs being discarded, but still allows for thousands of candidates for further studies.

One potential caveat for this type of discovery tool concerns the reproducibility of gene expression measurements. Array based measurements of gene expression are now commonplace, but in studies resulting in hundreds of millions of candidate relationships the precision of the data becomes especially critical. A comparison of gene expression studies of the NCI60 cell lines highlighted this issue by revealing a surprisingly low level of correspondence in expression measurements [25]. However, we show here that a novel gene expression data set across the NCI60 cell lines measured using spotted cDNA arrays is very reproducible in comparison to three publicly available datasets from independent labs using independent RNA preps. This is one of the largest available datasets measuring the overall reproducibility of gene expression data measured by gene arrays and demonstrates sufficient reproducibility. The higher degree of reproducibility shown here between datasets than other comparison studies likely results from a different metric of similarity being employed, and likely of greater importance, greater filtering of the datasets to remove genes apt to be poorly measured.

A comparison of compound sensitivity and gene expression profiles for the identification of candidate targeted lead compounds presupposes that the biological association between the compound and gene is strong. However gene expression studies on cell lines have been used to identify and explore broad physiological features of cell growth and responses for which multiple genes share similar expression patterns. For example, an earlier expression study using a smaller gene set on the NCI60 cell lines revealed a novel classification of the lines based on the tissue characteristics of the lines, into melanoma, leukemia, epithelial-like and mesenchymal-like categories [6]. The cell lines of this study were very similarly classified using a much larger gene set (data not shown). Therefore a correlation between a gene and a drug may indicate that the drug's metabolism or action is directly affected by the gene is question, or the effect may be secondary, the gene may be only weakly associated to the true critical biochemical pathways. Such secondary associations between drug and gene would not invalidate the screen as a tool for finding genes that might predict response to a specific, but do decrease the likelihood that the identified associations would remain tightly linked in systems beyond the cell lines in question. It would also decrease the likelihood that one could experimentally validate in cell line models the link between a drug to the expression of a specific gene. However, broad gene expression patterns as identified by principle component analysis contribute only moderately to gene-compound relationships in this study. The sole physiological feature that could be identified as contributing to some linkage of genes and drugs, the mesenchymal-like nature of some cell lines, is easily identifiable and these correlations ignored, if desired.

The gene expression and compound sensitivity data from the NCI60 cell lines represent a rich source of hypotheses that have still much to offer. However, the attempt to associate these two types of information is based on several assumptions, including that the molecular physiology of the cells determines their chemosensitivity, that mRNA levels are a robust means of characterizing the cell's molecular physiology, and that tumor cells lines are a valid model for solid tumors. Obviously these assumptions will not always be valid, the question remains the degree to which they are, and how readily does this screening approach produce candidates that validate in the clinic. That the approach can yield valuable leads has been shown by many anecdotal reports. We have established in this study that, on a global scale, the identified associations are reproducible and likely to be relatively specific. More work is needed to truly prove the worth of this type of analysis to the cancer patient.

## List of Abbreviations

GI50: 50% growth inhibition; GNF: Genomics Institute of the Novartis Research Foundation.

## Authors' contributions

BZR and DTR designed the experiments. BZR and DTR wrote the paper. SC carried out the gene expression assays. BZR, RSS, LWR, and DTR analyzed the data. All the authors have read and approved the final version of the manuscript.

## Supplementary Material

Additional file 1 Supplementery Table 1. Cell lines employed in these studies. The three groupings of cell lines, NCI7, NCI39, and NCI60 are detailed, as well as the tissue type to which the cell lines relate. Click here for file

Additional file 2 Gene Expression Data tables.xls. The gene expression data for the genes used in the analysis. The NCI60 and NCI7 data are supplied as separate worksheets. Click here for file

Additional file 3 GI50 Data tables.xls. The compound sensitivity data used in the analysis. The NCI60 and NCI7 data are supplied as separate worksheets. Click here for file

Additional file 4 NCI60GI50scorrs.txt. The correlations (|r| >= 0.5) between compound sensitivity and gene expression in the NCI60 cell lines. Click here for file
